# Histopathological Diagnostic Discordance between Punch Biopsies and Final Diagnostic Excisions of Cutaneous Squamous Cell Carcinoma: Analysis of 737 Cases

**DOI:** 10.2340/actadv.v105.40727

**Published:** 2025-01-03

**Authors:** Katharine HOPKINS, Åsa INGVAR, Johan PALMGREN, Valdis THORHALLSDOTTIR, Kari NIELSEN, Karim SALEH

**Affiliations:** Division of Dermatology and Venereology, Department of Clinical Sciences, Lund University, Skåne University Hospital, Lund, Sweden

**Keywords:** histopathology, puncture biopsy, sensitivity and specificity, squamous cell carcinoma

## Abstract

The recommended treatment for cutaneous squamous cell carcinoma is surgical excision. An initial punch biopsy is often performed as an aid to diagnosis. A retrospective registry-based study was performed to assess histopathological concordance of punch biopsy of cutaneous squamous cell carcinoma and subsequent excision. Analysis of 737 punch biopsies and subsequent matched excisions was performed. In total, 493 (67%) lesions were confirmed as invasive cutaneous squamous cell carcinoma on excision, 76% when excluding “scar” as a final diagnosis. Tumour diameter > 20mm was highly predictive of cutaneous squamous cell carcinoma (positive predictive value 91.1%). Tumours on the scalp were significantly more likely to demonstrate a final diagnosis of cutaneous squamous cell carcinoma than those on the arm (odds ratio 6.11, 95% confidence interval 3.1,12.0). There was moderate concordance between biopsy and excision in grade of histopathological differentiation. This study demonstrates that clinical high-risk features may be of more value in predicting a diagnosis of cutaneous squamous cell carcinoma than partial punch biopsy. Use of clinical and dermoscopic competencies in assessment of cutaneous tumours rather than reliance on biopsies both avoids delay in patient management in the case of high-risk cutaneous squamous cell carcinoma and may also minimize unnecessary surgical excisions if there is a low clinical index of suspicion of cutaneous squamous cell carcinoma.

## SIGNIFICANCE

Cutaneous squamous cell carcinoma is a common form of skin cancer and is increasing in prevalence. Often, partial biopsies are performed on suspected tumours to confirm the diagnosis before removal via an operation. This results in a delay in treatment while the results are awaited. Here we demonstrate that the biopsy is of limited value in confirming the diagnosis of squamous cell carcinoma. Tumours that were more likely to be squamous cell carcinoma based on clinical examination were more likely to be confirmed as such after final surgery. Biopsies can lead to both unnecessary excisions and delays in patient management.

Cutaneous squamous cell carcinoma (cSCC) is the second most common type of skin cancer in fair-skinned individuals, and accounts for 20% of keratinocyte carcinomas (1–3). It is a burden on healthcare systems, with incidence in the USA increasing 263% between the periods 1976–1984 and 2000–2010 (4). In Sweden, data show a year-on year increase in diagnosis of cSCC of 4.2% for men and 4.8% for women in the 10 years prior to 2019 (5). This rise in incidence can be explained by ageing populations and public awareness of skin cancer symptoms. Due to differences in cancer registration in different countries and low disease mortality, it is thought that the true incidence is widely underestimated. Although mortality is lower than for cutaneous melanoma, there are several implications of the disease: many patients have multiple lesions and tumours can be locally infiltrative and destructive, metastasize to regional lymph nodes, or cause distant metastasis. Rate of metastasis is low (2–5%) but has a poor prognosis with a median survival of less than 2 years (6). Therefore, prompt accurate diagnosis and care are of importance.

It is hypothesized that cSCC often lies at the end of a spectrum that encompasses premalignant actinic keratoses and squamous cell carcinoma in situ (7). Clinically, it can be difficult to differentiate between these lesions, and previous studies have shown that clinical examination has a low sensitivity of approximately 40% in determining diagnosis of cSCC (8, 9), although use of dermatoscopy as an aid to diagnosis has yielded promising results (10, 11). Currently, the gold standard method of obtaining a definitive diagnosis is histopathological examination.

The primary aim of this study was to assess whether there is benefit in obtaining punch biopsy of cSCC as an aid to diagnosis prior to surgical excision by assessing whether there is histopathological concordance between biopsy and subsequent surgical excision. The authors also aimed to investigate whether the presence of high-risk clinical parameters, such as tumour location, size, and degree of differentiation on initial biopsy may be useful tools in predicting whether a suspected lesion is a cSCC.

## METHOD

A retrospective observational registry-based study was performed in the county of Skåne, Southern Sweden, which has a population of approximately 1.5 million. Data were obtained on all biopsies performed by clinicians in the dermatology departments of the 7 public hospitals in the region, during the period 1 January 2015, and 31 December 2019, which were classified by the reporting pathologists as a cSCC. Patient and tumour characteristics were obtained from the patient’s electronic medical record system, where all biopsies and excisions as well as the histopathological reports are registered. If a patient had multiple tumours during the study period, all were included in the study and subsequent analyses. As the aim of the study was to assess real-world diagnosis and reporting, the histopathological slides were not re-reviewed or interrogated. In the study region, dermatological histological preparations are reported by dermatopathologists and pathologists with specialized expertise in dermatopathology. Patients under 18 years of age and biopsied lesions that were not followed by surgical excision were excluded. As the aim was to compare diagnostic punch biopsy data with subsequent excisional data all other biopsy types (shave, curettage) were excluded from further analyses. Size of punch biopsy is not routinely recorded in the medical record in Sweden and was therefore not included in analysis.

Patient and tumour-related factors including sex, age, tumour size, anatomical localization, histopathological degree of differentiation on biopsy, and final histopathological diagnosis after subsequent excision of the lesion were registered. The final degree of differentiation when the tumour was determined to be a cSCC was also recorded. It was agreed that, for the purposes of analysis, a final diagnosis of invasive cSCC of any grade would be defined as “concordant” with the initial biopsy, while any other diagnosis would be defined as “discordant”. Final diagnoses of keratoacanthoma were categorized as squamous cell carcinomas due to the complexities associated with clinical and histopathological interpretation of keratoacanthoma. The recorded histopathological degree of differentiation was divided into high risk (poor differentiation) and low risk (high, medium-high, not specified) (3).

Study data were recorded in an electronic database with personally identifiable information removed. Ethical approval for the study was obtained from the Swedish Ethical Review Authority (application number 2020-02895).

### Statistical analysis

Descriptive statistics were compiled on patient and tumour characteristics.

Concordance between punch biopsy result and final diagnosis was assessed using descriptive statistics. Final diagnosis of invasive cSCC of any grade was defined as “concordant” with the initial biopsy and “discordant” was used to define any other final diagnosis.

For analysis of tumour size and final histopathological diagnosis on excision, lesions were divided into 2 groups, “low risk” lesions with diameter ≤ 20 mm and “high risk” lesions with diameter > 20 mm. χ^2^ analysis was used to examine the relation between tumour size and final diagnosis of cSCC, and sensitivity, specificity, and positive predictive value (PPV) for initial tumour size and final histopathological diagnosis (cSCC or alternative diagnosis) were calculated.

For assessment of lesion location and final histopathological diagnosis the cases were divided into 6 groups according to anatomical site of the lesion (scalp, ear, face and neck, trunk, arm, and leg). Odds ratios (OR) for the different locations and final histopathological diagnosis were calculated with and without adjustment for lesion size using logistic regression analysis. The location of “arm” was used as the reference variable.

Inter-rater reliability between histologic parameters in biopsies and excisions was performed using cross-tabulations and Cohen’s kappa (κ) coefficient (interpretation of kappa result: ≤ 0 no agreement, 0.01–0.20 none to slight, 0.21–0.40 fair, 0.40–0.60 moderate, 0.61–0.80 substantial, and 0.81–1.00 almost perfect agreement). A *p*-value < 0.05 was considered statistically significant. All data analyses were performed with SPSS (Statistical Package for the Social Sciences) version 29 (IBM Corp, Armonk, NY, USA).

## RESULTS

### Cases

A total of 1,346 biopsies demonstrating cSCC were registered in the regional pathology registry during the study period. Of those, 926 (69%) were punch biopsies, 73 (5%) shave biopsies, and 347 (26%) curettage biopsies. After exclusions, 737 punch biopsies in 659 patients with subsequent excisional data were recorded during the study period. There were 274 female patients, with a total of 298 (40.4%) tumours, and 385 male patients, with a total of 439 (59.6%) tumours. The median age of included patients was 79.0 years.

### Final diagnosis

In total, 493 (67%) of the tumours reported as cSCC on the initial punch biopsy were reported as cSCC on the subsequent surgical excision. Thus, an alternative diagnosis was given in the subsequent excision in 33% of cases. Alternative diagnoses are displayed in **Table I** and included, for example, cSCC in situ (9.1%, 67 cases) and scar (12.3%, 91 cases). Excluding “scar” from the final diagnoses increased the diagnostic concordance of cSCC on biopsy and excision to 76% (493/646) (Table I).

**Table I:** Final diagnosis of lesions after operative excision

Final diagnosis	Number	Percentage
Squamous cell carcinoma	493	66.9
Scar	91	12.3
Squamous cell carcinoma in situ	67	9.1
Actinic keratosis	31	4.2
Basal cell carcinoma	19	2.6
Other	19	2.6
Benign	8	1.0
Metatypical	5	0.7
Adnexal tumour	2	0.3
Dermatitis	2	0.3
Total	737	100

### Tumour size

Information on initial tumour size was available in 712 cases of 737 (96%). The median tumour size was 11 mm (interquartile range 8–20 mm). A clinical diameter > 20 mm was seen in 123 (17.3%) tumours, and 112 of these were subsequently reported as a cSCC on excision. χ^2^ analysis demonstrated a significant association between tumour size and final diagnosis of cSCC, χ^2^ (1, *n* = 712) = 37.83, *p* < 0.00001.

The PPV of cSCC in tumours with a diameter > 20 mm was 91.1% (95% confidence interval [CI)] 84.8–94.9%). For tumour diameter ≤ 20 mm, the PPV of cSCC on excision was significantly lower at 62.5% (95% CI 61.1–63.8%) (**Table II**).

**Table II:** Initial tumour size and subsequent diagnosis, where tumour size was recorded

Size	Final diagnosis cSCC, *n* (%)	Other final diagnosis, *n* (%)	Total
Tumor diameter ≤ 20 mm	368 (62.5)	221 (37.5)	589
Tumor diameter > 20 mm	112 (91.1)	11 (8.9)	123
Total	480	232	712

cSCC: cutaneous squamous cell carcinoma.

### Anatomical site

The face and neck (294 lesions, 39.9%) and scalp (129, 17.5%) were the most common locations of biopsied lesions. Tumours on the scalp (122, 86.8%) and the ear (45, 73.8%) were most likely to be reported as cSCC on histopathological examination following subsequent excision. Logistic regression analysis assessing the effect of lesion location on final histopathological diagnosis demonstrated that the scalp was over 6 times as likely to give a final diagnosis of cSCC compared with the arm (OR = 6.11, 95% CI 3.1,12.0), and the ear was over twice as likely (OR = 2.61, 95% CI 1.3,5.4). Increased odds were also demonstrated for face and neck (OR = 1.87, 95% CI 1.1,3.2). These differences were maintained when adjusted for tumour size (**Table III**).

**Table III:** Illustration of frequency of cutaneous squamous cell carcinoma (cSCC) by anatomical location

Location	Biopsies, *n* (%)	Final diagnosis cSCC, *n* (%)	OR for final diagnosis cSCC		OR for final diagnosis cSCC, adjusted for tumour size	
			OR	95% CI for OR	OR	95% CI for OR
Arm	81 (11)	42 (51.9)				
Face and neck	294 (39.9)	194 (66.0)	1.80	1.1–3.0	1.87	1.1–3.2
Leg	106 (14.4)	59 (55.7)	1.16	0.7–2.1	1.01	0.5–1.9
Trunk	66 (9)	41 (62.1)	1.52	0.8–3.0	1.30	0.6–2.6
Ear	61 (8.3)	45 (73.8)	2.61	1.3–5.4	2.56	1.2–5.4
Scalp	129 (17.5)	122 (86.8)	6.11	3.1–12.0	5.56	2.7–11.4
Total	737	493				

### Grade of differentiation

Of the 493 cSCCs that were confirmed upon excision, the histopathological grade of differentiation was in agreement with the biopsy result in 438 cases (89%), giving a Cohen’s Kappa coefficient of 0.43 (moderate agreement) (*p* = 0.000, 95% CI 0.37–0.56). A final histopathological diagnosis of poorly differentiated cSCC was seen in 53 tumours, of which 26 (49%) were not identified on initial biopsy. In addition, 29/56 (51.8%) tumours identified as a poorly differentiated cSCC on biopsy were reclassified as a non-poorly differentiated cSCC on subsequent excision. Sensitivity of punch biopsy in identifying poorly differentiated cSCC was 50.9% (95% CI 36.8–64.9) (**Table IV**).

**Table IV:** Agreement between biopsy and excision regarding poor grade of differentiation

Factor		Poorly differentiated cSCC on excision, *n*		Total
		Yes	No	
Poorly differentiated cSCC on biopsy, *n*	Yes	27	29	56
	No	26	411	493
		53	440	

cSCC: cutaneous squamous cell carcinoma.

## DISCUSSION

Our results suggest that there is moderate correlation between histopathological reporting of cSCC identified on a punch biopsy and subsequent surgical excision. In total, 33.1% of lesions diagnosed as a cSCC on initial biopsy were reported as a different diagnosis after surgery, 23.7% if excluding “scar” as final diagnosis. In this study, the high-risk clinical parameters of tumour size > 20 mm and anatomical location of the head and neck region demonstrated significantly increased likelihood of the final diagnosis being a cSCC. Regarding histopathological grade of differentiation, punch biopsy had a low sensitivity in identifying poorly differentiated (high risk) cSCC.

European Guidelines state that histological confirmation should be performed in lesions that are clinically suspicious of cSCC (3) and that this can take the form of either a partial biopsy or an excisional biopsy of the entire lesion. In common clinical practice in Sweden, a punch biopsy is often used as a diagnostic aid if there is clinical uncertainty whether the lesion represents a cSCC or a preinvasive lesion, or to obtain preoperative information concerning the presence of high-risk histopathological features. It is thought that this may aid the decision-making process when determining excision margins and further management. Importantly, when technically possible, definitive management of a cSCC is surgical excision and, therefore, biopsy performed prior to excision could lead to potential delays in definitive surgical management.

There are multiple potential reasons for discrepancy between initial biopsy diagnosis and subsequent excision diagnosis of cSCC. First, we can hypothesize that the quality of biopsy material, which is decisive in classification of a lesion within the spectrum of actinic keratosis to cSCC, may depend on biopsy technique and the morphology and location of the lesion. Of course, partial biopsies may not be representative of the whole lesion, and as both biopsy and excision specimens are routinely histopathologically evaluated with “breadloaf” sectioning, only a minor part of the lesion is examined. This could therefore lead to a potential discrepancy in reporting. There is also potential for other “bystander” lesions to be included in both biopsies and excisions and subsequent reporting.

Furthermore, a biopsy could potentially excise the invasive cSCC within the lesion, leaving limited non-invasive changes such as SCCiS or AK, or even no residual disease, on the subsequent excision. Interestingly, 12.3% of our cases were reported as “scar” after excision, implying that the original lesion might have regressed or that the biopsy was “curative”. There are case reports of spontaneous regression of lesions classified histopathologically as cSCC, presumably by a yet undescribed immunological mechanism (12, 13). This could be triggered by the subsequent inflammatory response after biopsy (13), and many dermatologists are familiar with the phenomenon anecdotally. This study did not record the presence of any clinically residual lesion prior to surgical excision and, hence, no conclusions can be drawn on whether this could guide the management of lesions that disappeared clinically upon punch biopsy. Current guidelines do emphasize that all confirmed cSCCs should be treated with excision with appropriate margins (6, 14, 15).

Initial tumour size was found to be a good predictor of final diagnosis of cSCC in our study, with clinical tumour diameter > 20 mm giving a PPV of 91.1%. Larger tumours could yield more suitable tissue for histopatho-logical analysis, or bigger punch biopsies could have been performed in the larger tumours. Regardless, it is known that tumour size > 20 mm is the most important clinical risk factor regarding recurrence of cSCC, and has also been shown to be the most important risk factor in disease-specific death from cSCC (16). Therefore, clinical diameter is used as the defining criterion in the TNM criteria when distinguishing between a high- and a low-risk tumour, thus informing subsequent management (17). Furthermore, our findings demonstrated that biopsy from a high-risk clinical site was predictive of a final diagnosis of cSCC, irrespective of tumour size (Table III). We suggest therefore that planning for surgical management with appropriate margins of excision could be based on a high clinical index of suspicion of cSCC in conjunction with tumour size and site on initial clinical presentation, rather than performing a preoperative biopsy.

In the dermatology setting, clinicians’ confidence in clinical diagnosis of cSCC has been improved with the increasing use of dermoscopy as an aid to diagnosis, and certain dermoscopic features can be predictive of the histological grade of cSCC (10, 11). Utilizing this tool should enhance dermatologists’ ability in formulating a preoperative diagnosis and management plan. However, in settings where this expertise is not available, for example within primary care, preoperative biopsy may still have an important role.

The moderate at best concordance regarding histopathological grade of differentiation between punch biopsies and subsequent excision suggests that punch biopsy is not a reliable method for identifying certain high-risk histopathological features of cSCC. As many high-risk features (tumour thickness, desmoplasia, and perineural invasion) are not routinely reported on histopathological reports in Sweden, they were not assessed in this study. A previous paper in which pathologists reviewed concordance between punch biopsies and excision in cSCC, including high-risk features, concluded that punch biopsy was inaccurate in determining TMN status in 1 in 6 tumours, and that biopsy specimens are rarely performed at a sufficient depth to reliably assess tumour thickness (18). One can therefore conclude that one of the putative arguments for performing a pre-excision biopsy, namely further assessment of certain histopathological high-risk features, can potentially be discounted.

The authors acknowledge that this study has limitations that should be taken into consideration. Due to the study design, we were unable to assess the clinicians’ reasons for performing the initial punch biopsy; it is unknownwhether it was to obtain a diagnosis or to obtain information on high-risk features of a clinically diagnosed cSCC for formulating a subsequent treatment plan (for example excision margins). As the study was retrospective and registry-based, data for all lesion characteristics was not available. Histopathological slides were not reviewed independently for this study, and it is known that concordance among pathologists in evaluating cSCC is poor (19). We consider that the strengths of the study are the high number of cases that were assessed, and the use of real-world data reflecting daily clinical practice. As already discussed, extension of the study to include assessment of other high-risk histopathological features could be of further benefit.

Our findings demonstrate moderate concordance between an initial punch biopsy diagnosis of cSCC and the subsequent final diagnosis. We suggest that there is limited merit in performing a pre-excision biopsy to confirm diagnosis of cSCC if one has a high clinical index of suspicion (using knowledge of clinical high-risk features and dermoscopic competence) that a lesion is a cSCC, especially in the case of larger lesions and lesions located in high-risk areas. In the latter cases, performing an initial biopsy to confirm diagnosis can potentially lead to a delay in definitive surgical management of cSCC, potentially influencing prognosis and feasibility of surgery, especially in high-risk tumours. It follows that employment of this strategy could also minimize unnecessary surgical excisions if there is a low clinical index of suspicion of cSCC.
